# Unfermented Wine

**Published:** 1875-06

**Authors:** 


### UNFERMENTED WINE.

Passing thus through the whole breadth of Europe on one of its chief wine-growing belts, it seems as if the usual grape-beverage might be taken as a fair sample of the drinks which the people of any such region will extract from the vine. The question will occur, Do these communities use or know any drink bearing the name of wine which is not fermented, intoxicating drink? It becomes apparent, even to the eye, that all the wines consumed by high and low are diffusible stimulants, stirring the blood, exciting the nerves, and flushing the cheeks. It is found that, while differing in astringency, sourness, and flavor, the people's wine is invariably an alcoholic drink, apparently a little stronger than the strongest cider which was formerly made in New England, from which, in some instances, the taste can scarcely be distinguished. If there be any practice of preserving the unfermented juice of the grape, or preserving the grape to make it such as the excellent Mr. Delavan found in one solitary instance in Italy, it is a practice kept profoundly secret. And if there be any unfermented liquors sold and drank as "wines" in wine-making regions, they are liquors unknown to the hotels, the *cafes*, the restaurants, and the people who frequent them; and they are liquors not comprised in the *vin ordinaire* of the great vintage zone. Their existence is a secret undiscovered, and it is probable that unfermented wine is absolutely unknown throughout all wine-growing lands. It is a difficult matter to preserve the rich grape-juice from fermentation, and fermentation produces alcohol. Alcohol is, therefore, the first result of the death of the grape, the first stage of decomposition. It accompanies death, as a poison may well do.

